# Supplementary material for: Enhanced Mechanical Properties of Polylactic Acid/Poly(Butylene Adipate-co-Terephthalate) Modified with Maleic Anhydride
Source: Polymers (Basel). 2024 Feb 14;16(4):518. doi: 10.3390/polym16040518 (PMC10892572; doi:10.3390/polym16040518)
Supplement: Supplementary file 1 [file polymers-16-00518-s001.zip › polymers-2854696-supplementary.pdf]

polymers

Supporting_Information

Enhanced Mechanical Properties of Polylactic Acid/Poly(Butylene Adipate-co-Terephthalate) Modified with Maleic Anhydride

Kibeom Nam, Sang Gu Kim, Do Young Kim, Dong Yun Lee*

Department of Polymer Science and Engineering, Kyungpook National University, Daegu 41566, Republic of Korea

*Correspondence: dongyunlee@knu.ac.kr; Tel.: +82-53-950-5627; Fax: +82-53-950-6623

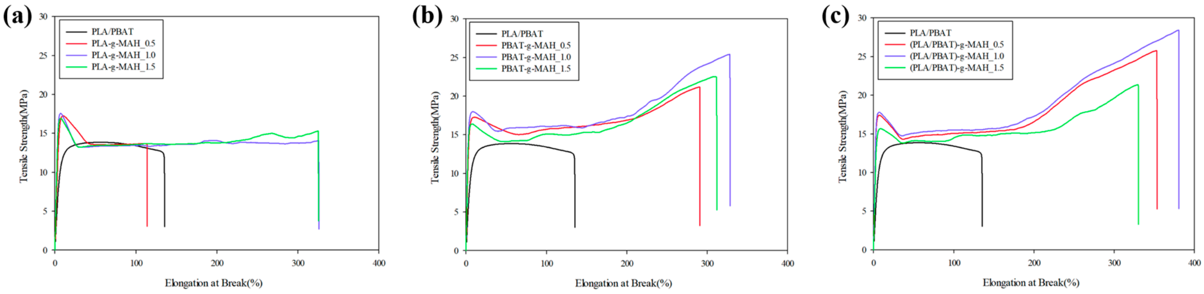

Figure S1. Stress–strain curves of (a) PLA-g-MAH/PBAT, (b) PLA/PBAT-g-MAH, and (c) (PLA/PBAT)-g-MAH.
